# Constitutive modeling of high temperature flow behavior in a Ti-45Al-8Nb-2Cr-2Mn-0.2Y alloy

**DOI:** 10.1038/s41598-018-23617-7

**Published:** 2018-04-03

**Authors:** Gengwu Ge, Laiqi Zhang, Jingjing Xin, Junpin Lin, Mark Aindow, Lichun Zhang

**Affiliations:** 1 State Key Laboratory for Advanced Metals and Materials, University of Science and Technology Beijing, Beijing, 100083 China; 2 Department of Materials Science and Engineering, Institute of Materials Science, University of Connecticut, Storrs, CT 06269-3136 USA

## Abstract

A constitutive equation based on the hyperbolic sinusoidal Arrhenius-type model has been developed to describe the hot deformation behavior of a β-γ Ti-Al alloy containing 8 at.% of Nb. Experimental true stress-true strain data were acquired from isothermal hot compression tests conducted across a wide range of temperatures (1273 K~1473 K) and strain rates (0.001 s^−1^~1 s^−1^), and the changes in the experimental conditions were reflected in the values of the Zener-Hollomon parameter. The impact of true strain was expressed through material constants (*A*, *α*, *n* and *Q*), and it was found that a 7th order polynomial is appropriate to express the relations between the true strain and these material constants. The average absolute relative error (*AARE*) and correlation coefficient (*R*) were used to evaluate the accuracy of the constitutive equation, and the values obtained were 6.009% and 0.9961, respectively. These results indicate that the type of constitutive equation developed here can predict the flow stress for this alloy with good accuracy over a wide range of experimental conditions. Thus, equations of this form could be applied more widely to analyses of hot deformation mechanism and microstructure evolution.

## Introduction

Intermetallic TiAl-based alloys have been considered as high temperature structural materials for a wide range of applications in the aerospace, automotive and energy industries, mainly because of their low densities, high melting temperatures, good high temperature strengths, high resistances to oxidation and excellent creep properties^1–3^. The exploitation of these attractive properties in industrial applications has been limited by the low ductilities and poor deformability that TiAl-based alloys exhibit at room temperature, although these shortcomings can be improved by microstructure optimization and alloy design^4,5^. TiAl-based alloys with high Nb contents have attracted increasing attention. The addition of Nb increases the melting point and ordering temperature of the alloy, which enables the service temperatures to exceed 900 °C, and these alloys exhibit both excellent oxidation resistances and creep strengths^6–8^. Meanwhile, the addition of Nb can increase the critical resolved shear stress and decrease the stacking fault energy, both of which inhibit dislocation glide, and so these alloys possess high strengths at elevated temperatures^9–12^.

Conventional TiAl alloys with high Nb contents contain mainly α_2_ and γ phases, and can only be forged under canned or isothermal conditions^13,14^. It has been reported that excellent hot-working characteristics can be achieved in such alloys if the volume fraction of the β phase is increased^15–17^. The presence of the disordered bcc β phase provides sufficient independent slip systems during the hot deformation process; this improves the deformation behavior and promotes dynamic recrystallization^18–21^. The β phase exhibits B2 order upon cooling to room temperature. This B2 phase is hard and brittle at lower temperatures thereby compromising the ductility, but it is soft at elevated temperatures giving reduced creep resistance^16,22^. These issues can be ameliorated by heat treatment after hot-working to decrease the volume fraction of the B2 phase^22–25^. As such, a better understanding of the hot deformation behavior, and the effects of subsequent heat treatment, is required if the full potential of these alloys is to be realized.

It is important to understand the flow behavior of β-γ TiAl alloys with high Nb contents so that one can control the microstructure and mechanical properties of the workpiece during the hot working process^16,26^. Generally, the flow behavior of materials under various hot-working conditions can be described by constitutive equations, which accurately correlate the non-linear relationships between flow stress, strain, strain rate and temperature. Sellars and McTegart proposed a hyperbolic sinusoidal Arrhenius-type form for such equations, which adapt readily to a wide range of flow stresses^27,28^. The Zener-Hollomon parameter introduced into such Arrhenius-type equations is used to express the relation between the strain rate and the temperature, and the impact of true strain is expressed through material constants as functions of true strain. As such, hyperbolic sinusoidal Arrhenius-type constitutive equations can accurately predict flow stress values under a wide variety of hot deformation conditions. There have been previous attempts to apply constitutive equations of this type to TiAl-based alloys. For example, in studies by Gupta *et al*. on the hot deformation of alloys with 48% Al, 2% Nb and 2% Cr a constitutive equation was obtained and a processing map was constructed^29^. Similar studies were performed by Li *et al*. on near γ-phase alloys with 45% Al, 7% Nb and 0.2% W^26^. However, in these and most other studies on TiAl-based alloys, the constitutive equations were established based on peak flow stress values at a given strain, with little attention being paid to the effects of the strain on the material parameters. This limits the overall accuracy of such equations and restricts their application to the specific strain level(s) at which the original data were obtained. In this paper, we consider the hot deformation behavior of a β-γ Ti-Al alloy with 45% Al, 8% Nb, 2% Cr, 2% Mn and 0.2% Y. The experimental data is used to develop a constitutive equation in which the impact of true strain is incorporated using the strain iteration method. It is shown that the constitutive equation developed here can predict the flow stress accurately over a wide range of strain values. Thus, the equation covers alloys that have remarkable differences between peak and steady-state flow stress values. As a result, equations of this type can accurately describe the dynamic response of the hot deformability to the deformation parameters.

## Results and Discussion

### Microstructure and hot deformation behavior

**Figure 1:**
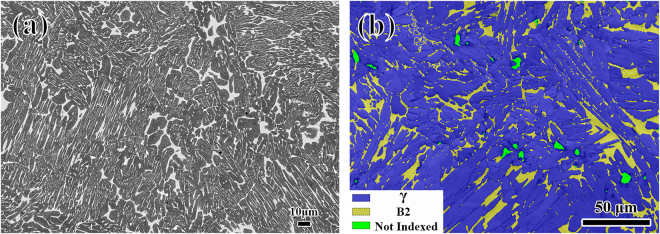
The as-cast microstructure of the β-γ alloy Ti-45Al-8Nb-2Cr-2Mn-0.2Y: (**a**) BSE SEM image (**b**) EBSD phase map.

Representative examples of a backscattered electron (BSE) SEM image and an EBSD phase map obtained from the as-cast microstructure are shown in Fig. 1. The initial microstructure exhibits mixtures of γ (gray) and B2 (bright) phases, and the identities of these phases were confirmed using the EDXS and EBSD data obtained from these regions. The EBSD phase maps indicate that the volume fraction of the B2 phase is about 11.4%.

**Figure 2:**
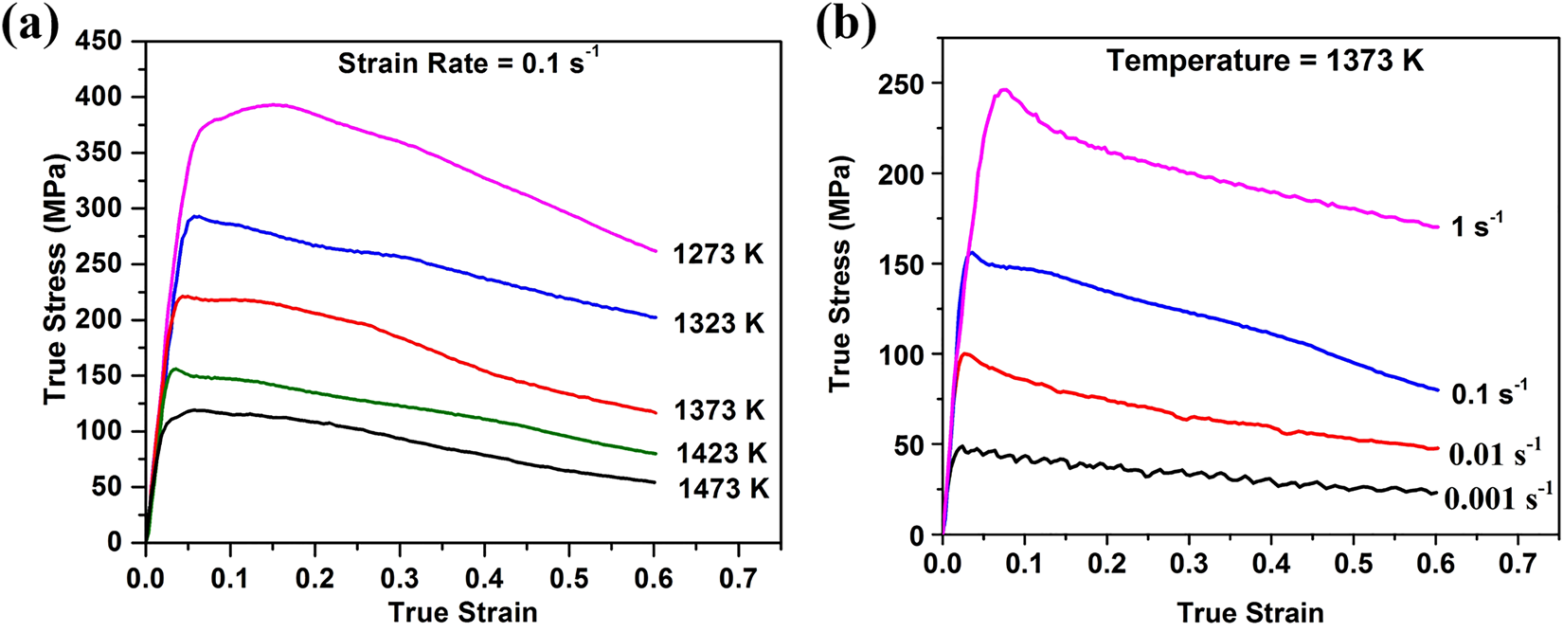
Examples of true stress – true strain curves obtained from the alloy: (**a**) at a fixed strain rate and different deformation temperatures, and (**b**) at a fixed temperature and different strain rates.

Examples of the experimental flow stress curves obtained from this alloy are presented in Fig. 2. These include curves for tests at each of the five testing temperatures for $\dot{\varepsilon }$ = 0.1 s^−1^ (Fig. 2(a)) and at each of the strain rates at T = 1423 K (Fig. 2(b)). Most of flow stress curves obtained in this study exhibit a single stress peak at relatively low strain ($\varepsilon \,$< 0.1), followed by a decrease in flow stress with increasing strain due to dynamic recrystallization. The values of the flow stress increase with decreasing deformation temperature at a given strain rate and with increasing strain rate at a given temperature (e.g. Fig. 2(a,b), respectively). Such behavior is expected because higher temperatures lead to increased dislocation mobilities, whereas lower strain rates allow more time for dynamic recrystallization processes to occur^20,30^.

### Constitutive analysis

#### Constitutive equation for flow stress prediction

Following the work of Zener and Hollomon^31^, the relation between the strain rate and the temperature during the hot deformation of metallic materials takes the form:

1 $$Z=\dot{\varepsilon }\exp (Q/{\rm{R}}T)$$

where: *Z* is the Zener–Hollomon parameter, representing the strain rate factor compensated by temperature; $\dot{{\rm{\varepsilon }}}$ is the strain rate (s^−1^); *Q* is the apparent activation energy of hot deformation (J·mol^−1^), R is the universal gas constant (8.314 J·mol^−1^·K^−1^), and *T* is the deformation temperature (K).

The correlation between flow stress ($\sigma$), *T* and $\dot{\varepsilon }$ for high temperature deformation, can be described by an Arrhenius-type constitutive model^26^. The relationship between the flow stress and the deformation parameters can be represented by the Zener-Hollomon parameter Z:

2 $$Z={\rm{F}}(\sigma )=\{\begin{array}{c}{A}_{1}{\sigma }^{{n}_{1}}\\ {A}_{2}\exp (\beta \sigma )\\ A{[\sinh (\alpha \sigma )]}^{n}\end{array}\,\begin{array}{c}\text{for}\,\alpha \sigma  < 0.8\\ \text{for}\,\alpha \sigma  > 1.2\\ \text{for}\,\text{all}\,\sigma \end{array}$$

where: *A*_1_, *A*_2_, *A*, *α*, *n*_1_, *n* and *β* are material constants; $\sigma$ is the flow stress (MPa), *α* is a parameter regulating the stress (MPa^−1^); and *n*_1_ and *n* are stress exponents. *α*, *β* and *n*_1_ are related by *α* = /*n*_1_.

Combining Eq. (1) and Eq. (2), we obtain three different Arrhenius-type equations that are applicable under different conditions. The simple exponential in Eq. (3) applies at low stress levels, the power exponential in Eq. (4) applies at high stress levels, and the hyperbolic sinusoidal Arrhenius-type Eq. (5) is suitable for calculating and characterizing hot deformation behaviors across the entire stress range.

3 $$\dot{\varepsilon }\exp (Q/{\rm{R}}T)={A}_{1}{\sigma }^{{n}_{1}}\,\begin{array}{c}\text{for}\,\alpha \sigma  < 0.8\end{array}$$

4 $$\dot{\varepsilon }\exp (Q/{\rm{R}}T)={A}_{2}\exp (\beta \sigma )\,\text{for}\,\alpha \sigma  > 1.2$$

5 $$\dot{\varepsilon }\exp (Q/{\rm{R}}T)=A{[\sinh (\alpha \sigma )]}^{n}\,\text{for}\,\text{all}\,\sigma $$

#### Determination of materials constants

The material constants for Arrhenius-type behavior can be determined using flow stress data from compression tests at different temperatures and strain rates. The true stress values for the alloy considered here change with increasing true strain, so the relations between the material constants and the true strain were evaluated at different values of the true strain. Below we demonstrate this process by showing examples of the calculations for the material constants at a true strain of 0.2.

For low or high stress levels, we obtain the following formulae by taking the natural logarithms of both sides of Eq. (3) and Eq. (4), respectively:

6 $$\mathrm{ln}\,\dot{\varepsilon }=\,\mathrm{ln}\,{A}_{1}+{n}_{1}\,\mathrm{ln}\,\sigma -\frac{Q}{{\rm{R}}T}$$

7 $$\mathrm{ln}\,\dot{\varepsilon }=\,\mathrm{ln}\,{A}_{2}+\beta \sigma -\frac{Q}{{\rm{R}}T}$$

**Figure 3:**
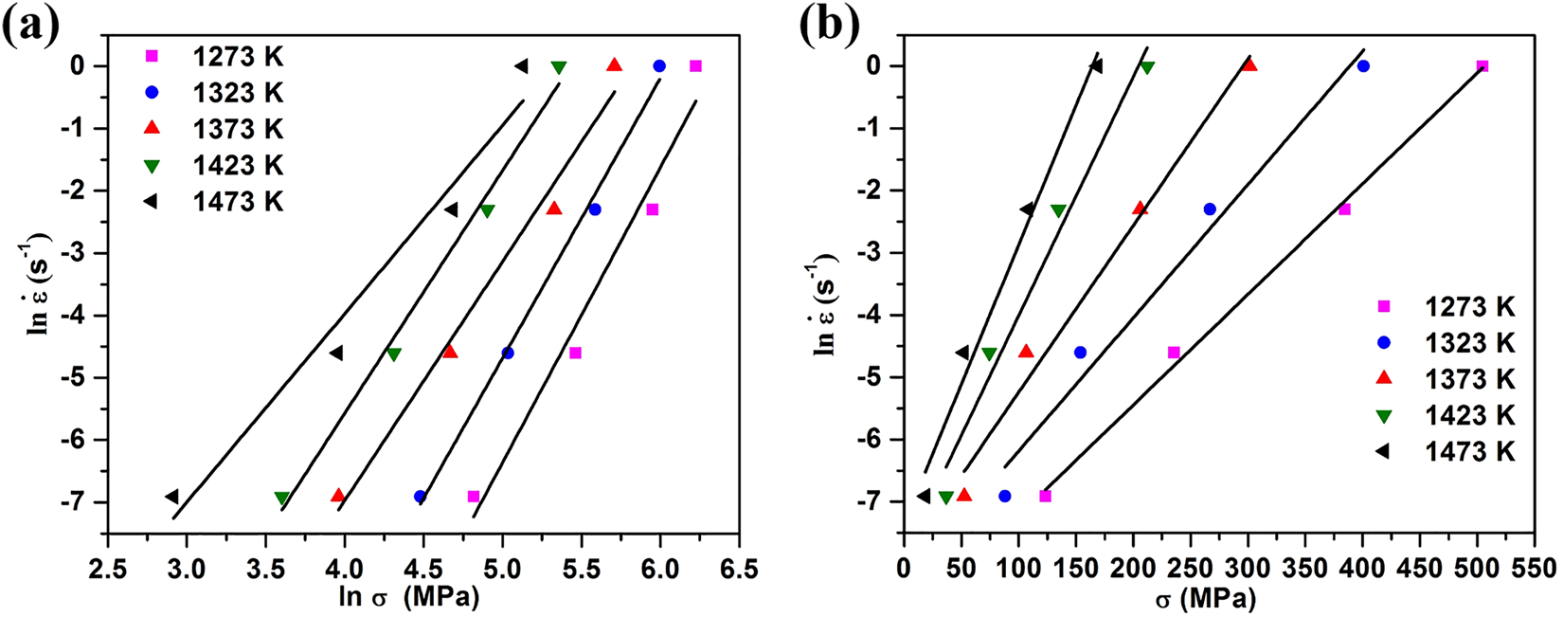
The relationships (as a function of temperature) between: (**a**) ln $\dot{\varepsilon }$ and ln*σ*, (**b**) ln $\dot{\varepsilon }$ and *σ*.

The values of *n*_1_ and *β* can be determined from the slopes of the $\mathrm{ln}\,\dot{{\rm{\varepsilon }}}-\,\mathrm{ln}\,\sigma$ plot and the $\mathrm{ln}\,\dot{\varepsilon }-\sigma$ plot, respectively at each temperature. Figure 3 (a,b) are the corresponding plots for values measured at a true strain of 0.2. The lines shown are least squares linear regression fits to the data. For the $\mathrm{ln}\,\dot{{\rm{\varepsilon }}}-\,\mathrm{ln}\,\sigma$ plot in Fig. 3(a), the slopes of the lines are very similar, particularly at the higher temperatures. The mean value of *n*_1_ obtained from the linear fits to the data at 1373 K, 1423 K and 1473 K is 3.5868. For the $\mathrm{ln}\,\dot{\varepsilon }-\sigma$ plot in Fig. 3(b), there is more significant variation in the slope, particularly at the higher temperatures, but the mean value of *β* obtained from the linear fits to the data at 1273 K, 1323 K and 1373 K is 0.0220. Using these values for *n*_1_ and *β* we obtain $\alpha =\beta /{n}_{1}$ = 0.0061.

For the entire stress range, we obtain the following formula by taking the natural logarithms of both sides of Eq. (5):

8 $$\mathrm{ln}[\sinh (\alpha \sigma )]=\frac{\mathrm{ln}\,\dot{\varepsilon }}{n}+\frac{Q}{n{\rm{R}}T}-\frac{\mathrm{ln}\,A}{n}$$

**Figure 4:**
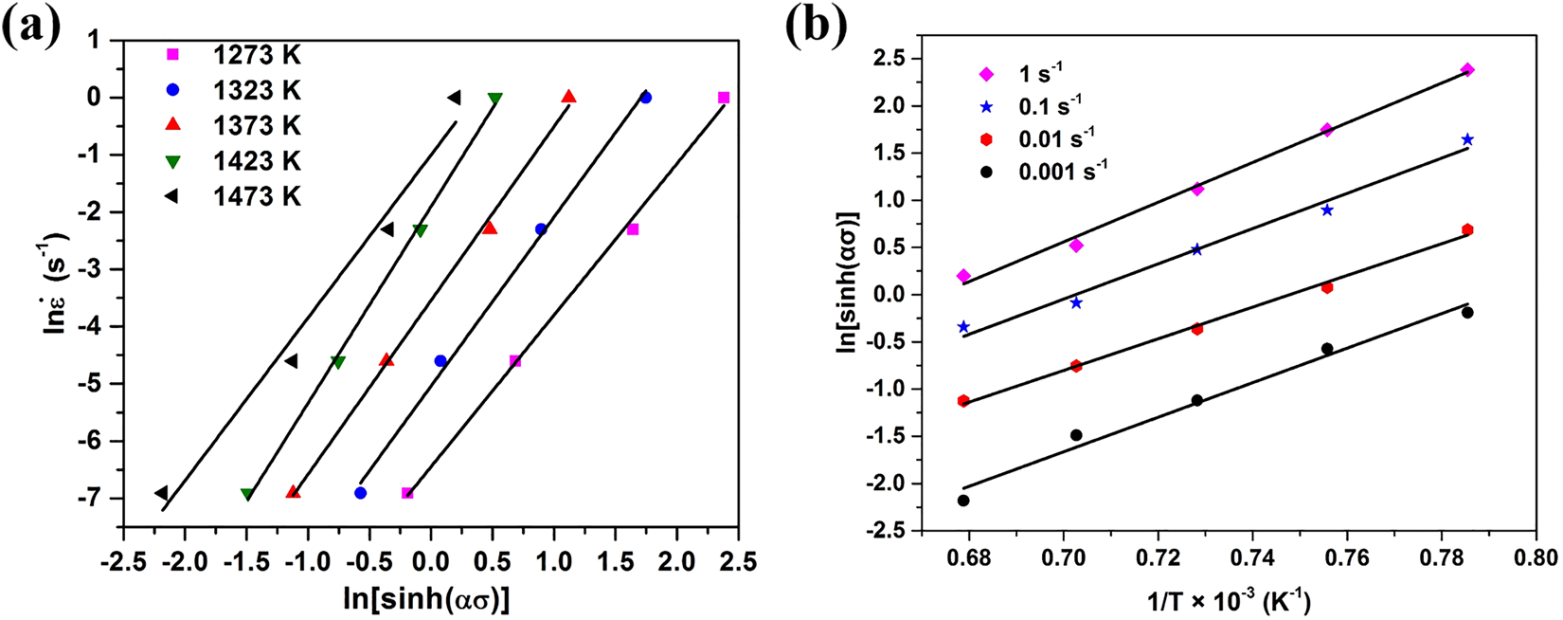
The relationship between (**a**) $\mathrm{ln}\,\dot{\varepsilon }$ and $\mathrm{ln}[\sinh (\alpha \sigma )]$ as a function of temperature, and (**b**) $\mathrm{ln}[\sinh (\alpha \sigma )]$ and 1/T as a function of strain rate.

According to Eq. (8), the value of n would be the mean value of the slopes for $\mathrm{ln}[\sinh (\alpha \sigma )]-\,\mathrm{ln}(\dot{\varepsilon })$ plots at different temperatures.Figure 4(a) is the corresponding plot for values measured at a true strain of 0.2, and these data give $n={\{\frac{\partial \mathrm{ln}\dot{\varepsilon }}{\partial \mathrm{ln}[\sinh (\alpha \sigma )]}\}}_{T}=2.9758$ for this alloy.

Moreover, if we take the logarithm and partial derivatives of both sides of Eq. (5), the following formula can be obtained for the activation energy.

9 $$Q={\rm{R}}{\{\frac{\partial \mathrm{ln}\dot{\varepsilon }}{\partial \mathrm{ln}[\sinh (\alpha \sigma )]}\}}_{T}{\{\frac{\partial \mathrm{ln}[\sinh (\alpha \sigma )]}{\partial (1/T)}\}}_{\dot{\varepsilon }}$$

Using the value of n acquired above and the mean value of the slopes for plots of $\mathrm{ln}[\sinh (\alpha \sigma )]-1/T$ at different strain rates (shown in Fig. 4(b) for values measured at a true strain of 0.2), the value of *Q* obtained using with Eq. (9) is 463.05 kJ·mol^−1^.

According to Eq. (8), the value of the fitted line intercept of $\mathrm{ln}(\dot{\varepsilon })-\,\mathrm{ln}[\sinh (\alpha \sigma )]$ equals $\frac{Q}{n{\rm{R}}T}-\frac{\mathrm{ln}\,A}{n}$, and the value of ln*A* at different temperatures can be obtained by from *Q*, *n*, *R* and *T*. The mean value of ln*A* obtained by this approach is 37.0911, and thus the material constant *A* = $1.2837\times {10}^{16}$. In this manner, all of the material constants required for the constitutive equation can be determined at a fixed value of true strain (0.2 in this case).

**Figure 5:**
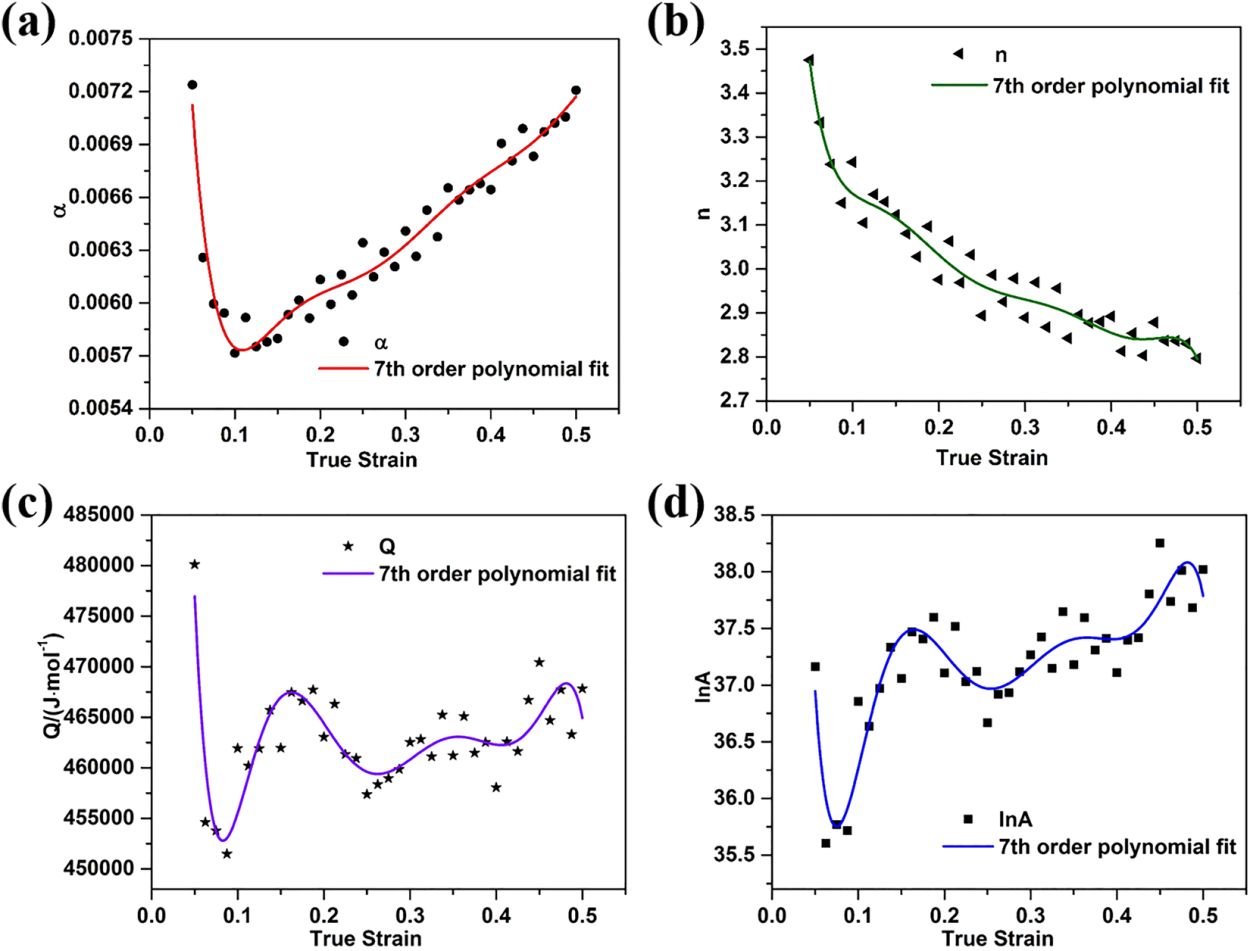
Variation of the material parameters (**a**) *α*, (**b**) *n*, (**c**) *Q* and (**d**) ln *A* with true strain.

**Figure 6:**
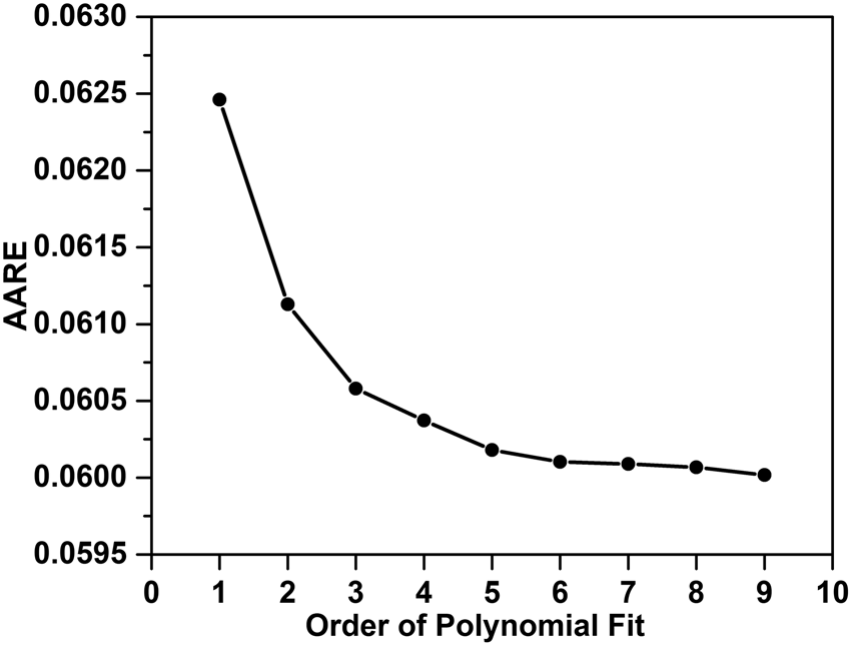
The relationship between *AARE* and order of polynomial fit.

To evaluate the effects of strain on the material constants *α*, *n*, *Q* and *A*, the values of these constants were obtained following the procedure described above for true strain values from 0.05 to 0.5 in increments of 0.0125. The values obtained for *α*, *n*, *Q* and ln*A* are shown in Fig. 5(a–d), respectively. Polynomial expressions were used to fit the variation with true strain in each case, and the degree of fit was evaluated by calculating values of the average absolute relative error (*AARE* – defined later in Eq. 15) for polynomials of different order from 1 to 9. As shown in Fig. 6, the value of *AARE* decreased with increasing polynomial order up to around 7th order polynomials, but thereafter there was little further improvement. Thus the 7th order polynomials shown in Eqs (10–13) were used to fit the plots in Fig. 5(a–d), respectively, and the values of the polynomial coefficients used are given in Table 1.

10 $$\alpha ={B}_{0}+{B}_{1}\varepsilon +{B}_{2}{\varepsilon }^{2}+{B}_{3}{\varepsilon }^{3}+{B}_{4}{\varepsilon }^{4}+{B}_{5}{\varepsilon }^{5}+{B}_{6}{\varepsilon }^{6}+{B}_{7}{\varepsilon }^{7}$$

11 $$n={C}_{0}+{C}_{1}\varepsilon +{C}_{2}{\varepsilon }^{2}+{C}_{3}{\varepsilon }^{3}+{C}_{4}{\varepsilon }^{4}+{C}_{5}{\varepsilon }^{5}+{C}_{6}{\varepsilon }^{6}+{C}_{7}{\varepsilon }^{7}$$

12 $$Q={D}_{0}+{D}_{1}\varepsilon +{D}_{2}{\varepsilon }^{2}+{D}_{3}{\varepsilon }^{3}+{D}_{4}{\varepsilon }^{4}+{D}_{5}{\varepsilon }^{5}+{D}_{6}{\varepsilon }^{6}+{D}_{7}{\varepsilon }^{7}$$

13 $$\mathrm{ln}\,A={E}_{0}+{E}_{1}\varepsilon +{E}_{2}{\varepsilon }^{2}+{E}_{3}{\varepsilon }^{3}+{E}_{4}{\varepsilon }^{4}+{E}_{5}{\varepsilon }^{5}+{E}_{6}{\varepsilon }^{6}+{E}_{7}{\varepsilon }^{7}$$

**Table 1:** Coefficients of the polynomial for $\alpha$, *n*, *Q* and ln *A*.

${\boldsymbol{\alpha }}$	*n*	*Q*	ln *A*
*B*_0_ = 0.01504	*C*_0_ = 5.40602	*D*_0_ = 780159.18855	*E*_0_ = 58.37288
*B*_1_ = −0.28833	*C*_1_ = −75.02224	*D*_1_ = −12477439.51656	*E*_1_ = −915.09628
*B*_2_ = 3.53849	*C*_2_ = 1034.47878	*D*_2_ = 183441948.28597	*E*_2_ = 14051.80978
*B*_3_ = −22.42069	*C*_3_ = −7519.762912	*D*_3_ = −1347235461.43665	E_3_ = −105888.32886
*B*_4_ = 80.47457	*C*_4_ = 30671.72251	*D*_4_ = 5459575900.15619	*E*_4_ = 436831.26062
*B*_5_ = −164.62043	*C*_5_ = −70662.79429	*D*_5_ = −12413953428.35000	*E*_5_ = −1006776.93325
*B*_6_ = 178.82574	*C*_6_ = 85831.304514	*D*_6_ = 14845336681.10690	*E*_6_ = 1217108.64917
*B*_7_ = −80.02126	*C*_7_ = −42705.11642	*D*_7_ = −7267633290.95133	*E*_7_ = −601273.11487

Thus, using a hyperbolic sinusoidal Arrhenius-type model, a constitutive equation expressing flow stress in terms of the Zener-Hollomon parameter can be written in following form (considering Eqs (1) and (5)):

14 $$\sigma =\frac{1}{\alpha }\,\mathrm{ln}\{{(\frac{Z}{A})}^{\frac{1}{n}}+{[{(\frac{Z}{A})}^{\frac{2}{n}}+1]}^{\frac{1}{2}}\}=\frac{1}{\alpha }\,\mathrm{ln}\{{(\frac{\dot{\varepsilon }\exp (\frac{Q}{{\rm{R}}T})}{A})}^{\frac{1}{n}}+{[{(\frac{\dot{\varepsilon }\exp (\frac{Q}{{\rm{R}}T})}{A})}^{\frac{2}{n}}+1]}^{\frac{1}{2}}\}$$

#### Analysis of constitutive equation accuracy

**Figure 7:**
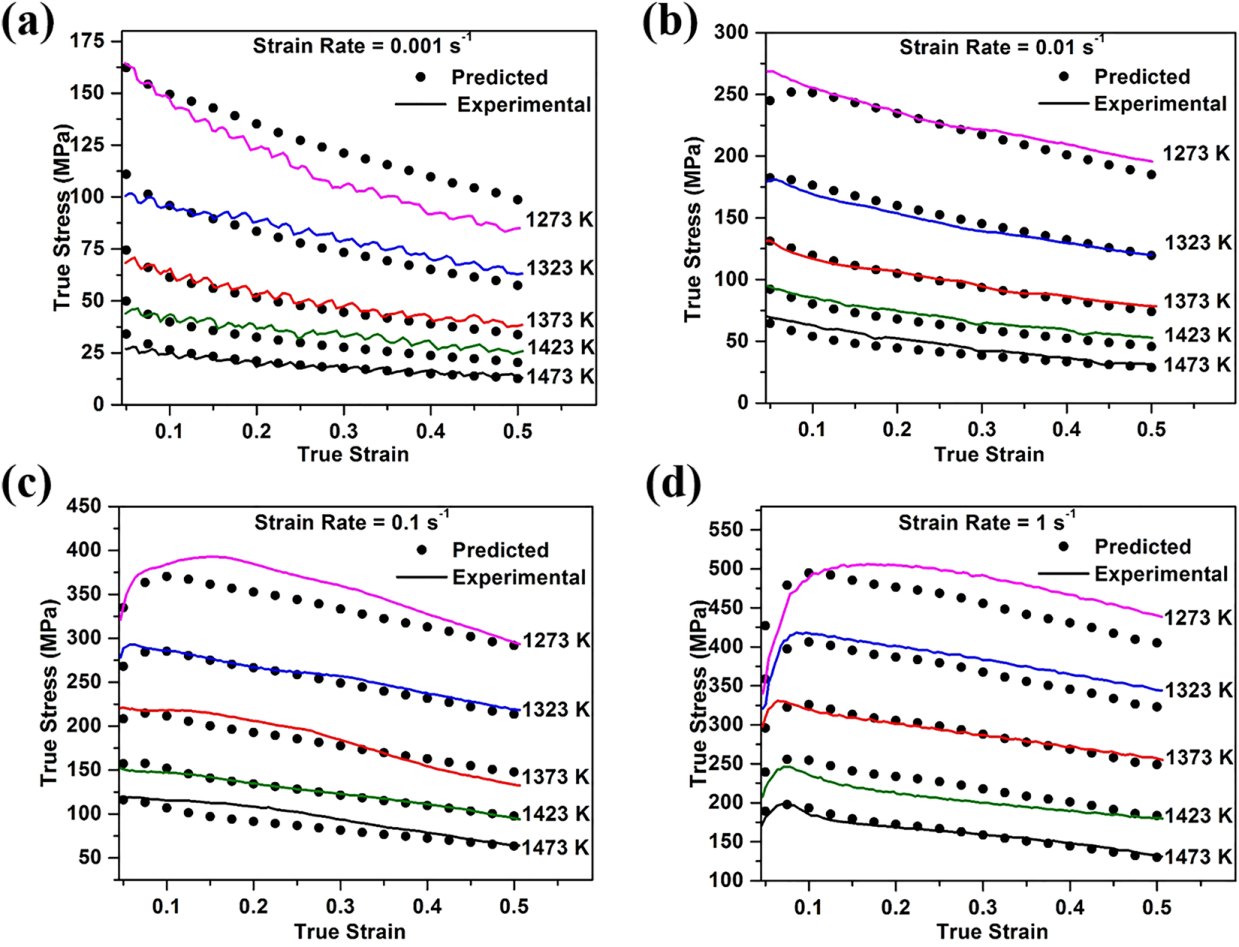
Comparison between the predicted and experimental flow stress at strain rate of (**a**) 0.001 s^−1^ (**b**) 0.01 s^−1^ (**c**) 0.1 s^−1^ and (**d**) 1 s^−1^.

The accuracy of the constitutive equation developed above can be evaluated by comparing the experimental and predicted flow stress values for the alloy considered in this study. The flow stress data obtained at different temperatures, strain rates and strains (in the range of 0.05 to 0.5 in increments of 0.025) were analyzed, and the predicted and experimental flow stress plots are presented in Fig. 7.

There is a good qualitative match between the experimental and predicted data under most conditions, but there are significant deviations under specific conditions. These are most notable for tests performed at 1273 K with strain rates of 0.001, 0.1 and 1 s^−1^. Such deviations have been noted previously, for example in studies on Ti-6Al-4V by Cai *et al*.^32^ and in studies on Ti-modified austenitic stainless steel by Mandal *et al*.^33^. One possible source for these deviations between the predicted and experimental data is the fitting of the material constants. Eqs (6) and (7) apply to low and high stress levels, respectively, and the approach used here is to obtain values for *n*_1_ and *β* from the mean slopes of the corresponding $\mathrm{ln}\,\dot{{\rm{\varepsilon }}}-\,\mathrm{ln}\,\sigma$ and $\mathrm{ln}\,\dot{\varepsilon }-\sigma$ plots. Thus, small errors could arise due to the accuracy of the least squares fit and/or differences in the slopes for different temperatures (see, for example, Fig. 3). Such errors may influence the values obtained for *α*, *n*, *Q* and *A*, and could therefore decrease the accuracy of the constitutive equation under some circumstances.

**Figure 8:**
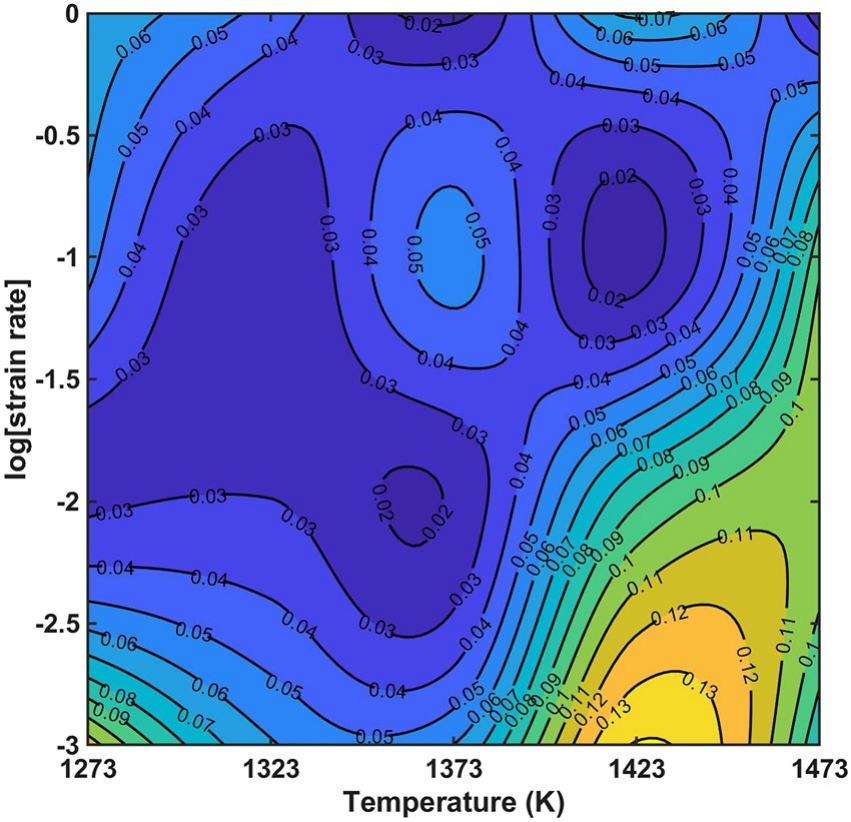
Distribution of *AARE* at temperature (1273~1473 K) and strain rate (0.001~1 s^−1^).

**Figure 9:**
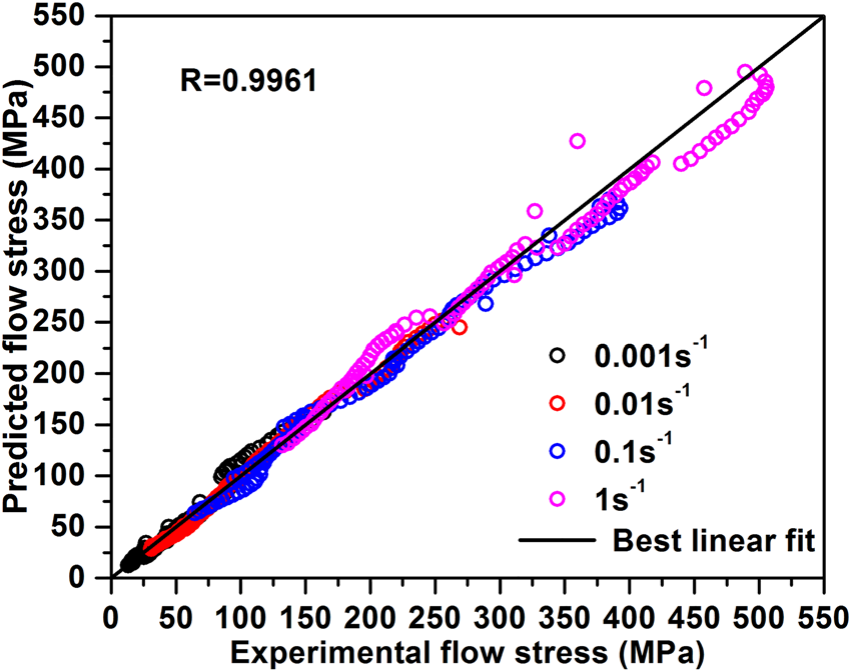
Comparison between predicted and experimental data.

To evaluate the degree of fit between a constitutive equation and an experimental data set quantitatively, it is usual practice to employ standard statistical parameters such as the average absolute relative error (*AARE*) and the correlation coefficient (*R*) (see, for example,^34,35^). These parameters are defined as:

15 $$AARE( \% )=\frac{1}{N}\sum _{i=1}^{N}|\frac{{E}_{i}-{P}_{i}}{{E}_{i}}|\times 100$$

16 $$R=\frac{{\sum }_{i=1}^{N}({E}_{i}-\bar{E})({P}_{i}-\bar{P})}{\sqrt{{\sum }_{i=1}^{N}{({E}_{i}-\bar{E})}^{2}{\sum }_{i=1}^{N}{({P}_{i}-\bar{P})}^{2}}}$$

where: *E* is the experimental flow stress; *P* is the predicted flow stress obtained from the constitutive equation; *N* is the quantity of selected data; and $\bar{E}$ and $\bar{P}$ are mean values of *E* and *P* respectively. Figure 8 is a contour plot of *AARE* values as a function of T and log $\dot{\varepsilon }$ over the range of experimental parameters considered in this study. The peak value of *AARE* is 14.07% at T = 1423 K and $\dot{\varepsilon }$ = 0.001 s^−1^. The values of *AARE* are significantly lower for $\dot{\varepsilon }$ of 0.01 to 1 s^−1^. The cumulative *AARE* is 6.009%, which shows that the constitutive equation is a good overall fit to the experimental data. Another measure of this is shown in Fig. 9, which is a direct plot of experimental against predicted flow stress values for all of the experimental parameters considered in this study. The correlation coefficient for the least squares fit line through the data is *R* = 0.9961, which demonstrates that there is a good linear relationship between the experimental and predicted values.

## Conclusions

1. Experimental flow stress data have been obtained from as-cast samples of a β-γ alloy with a composition of Ti-45Al-8Nb-2Cr-2Mn-0.2Y (all in at.%) by performing isothermal hot compression tests, across a wide range of temperatures (1273–1473 K) and strain rates (0.001–1 s^−1^). In each case, the flow stress increases with decreasing deformation temperature at a fixed strain rate and with increasing strain rate at a fixed temperature.
2. An Arrhenius-type constitutive equation for the hot deformation was established for true strain values ranging from 0.05 to 0.5. Values of the material constants (*A*, *α*, *n* and *Q*) in the constitutive equation have been determined as functions of the true strain. It has been shown that 7^th^-order polynomials are suitable to express the effect of strain on the material constants with acceptable *AARE*.
3. A comparison of the experimental data with the values predicted data using the constitutive equation gives values for *AARE* and *R* of 6.009% and 0.9961, respectively. These measures confirm that the hyperbolic sinusoidal Arrhenius-type constitutive equation developed here is a good model for predicting the hot deformation characteristics of β-γ Ti-Al alloys of the type considered in this study.

## Materials and Methods

The nominal chemical composition of the β-γ Ti-Al alloy used in this study was Ti-45Al-8Nb-2Cr-2Mn-0.2Y (all in at.%). An ingot of this alloy was produced by the vacuum levitation melting. Cylindrical specimens of 15 mm in height and 8 mm in diameter were cut from the as-cast ingot. Isothermal hot compression tests were conducted using a Gleeble-1500D thermo-mechanical simulator. Each of the compression tests were performed at a constant temperature (T = 1273, 1323, 1373, 1423 and 1473 K) and strain rate ($\dot{\varepsilon }$ = 1, 0.1, 0.01 and 0.001 s^−1^). In each case, the tests were terminated at a final reduction in height of 50%. To reduce the effects of experimental errors due to friction, the top and bottom surfaces of the specimens were polished before testing, and graphite foils were placed between the anvils and the polished specimen surfaces during the tests.

The specimens for electron back-scatter diffraction (EBSD) analysis were prepared by mechanical polishing, followed by electro-polishing using a solution of 5 vol.% perchloric acid, 30 vol.% butanol and 65 vol.% methanol under conditions of 253 K and 30 V. The samples were examined in a Zeiss Supra 55 scanning electron microscope (SEM) equipped with a HKL fast acquisition EBSD system and an Oxford X-Max energy-dispersive X-ray spectrometer (EDXS). The EBSD data were acquired over a grid of points at an interval of 0.25 μm, and the data were analyzed using HKL Channel 5 software.
